# HopDock: a probabilistic search algorithm for decoy sampling in protein-protein docking

**DOI:** 10.1186/1477-5956-11-S1-S6

**Published:** 2013-11-07

**Authors:** Irina Hashmi, Amarda Shehu

**Affiliations:** 1 Department of Computer Science, George Mason University, 4400 University Dr., Fairfax, VA, 22030, USA; 2 Department of Bioengineering, George Mason University, 4400 University Dr., Fairfax, VA, 22030, USA; 3 School of Systems Biology, George Mason University, 10900 University Blvd., Manassas, VA, 20110, USA

## Abstract

**Background:**

Elucidating the three-dimensional structure of a higher-order molecular assembly formed by interacting molecular units, a problem commonly known as docking, is central to unraveling the molecular basis of cellular activities. Though protein assemblies are ubiquitous in the cell, it is currently challenging to predict the native structure of a protein assembly in silico.

**Methods:**

This work proposes HopDock, a novel search algorithm for protein-protein docking. HopDock efficiently obtains an ensemble of low-energy dimeric configurations, also known as decoys, that can be effectively used by ab-initio docking protocols. HopDock is based on the Basin Hopping (BH) framework which perturbs the structure of a dimeric configuration and then follows it up with an energy minimization to explicitly sample a local minimum of a chosen energy function. This process is repeated in order to sample consecutive energy minima in a trajectory-like fashion. HopDock employs both geometry and evolutionary conservation analysis to narrow down the interaction search space of interest for the purpose of efficiently obtaining a diverse decoy ensemble.

**Results and conclusions:**

A detailed analysis and a comparative study on seventeen different dimers shows HopDock obtains a broad view of the energy surface near the native dimeric structure and samples many near-native configurations. The results show that HopDock has high sampling capability and can be employed to effectively obtain a large and diverse ensemble of decoy configurations that can then be further refined in greater structural detail in ab-initio docking protocols.

## Background

Proteins do not operate in isolation. They achieve their biological function by interacting with one or more molecules to form higher-order assemblies. Structural characterization of protein assemblies (formed by interacting protein units) is central to understanding molecular interactions, designing new effective drugs, and unraveling the molecular basis for different chemical processes in the healthy or diseased cell [1].

There are mainly two predominant experimental techniques to elucidate the biologically-active structure of a protein assembly: X-ray Crystallography and Nuclear Magnetic Resonance (NMR). These techniques are time- and labor-intensive and are often limited by the size of the molecular assembly [2]. The number of protein-protein assemblies with structures deposited in the Protein Data Bank (PDB) [3] is small compared to that of single protein chains. Due to the biological importance and ubiquity of protein-protein assemblies and current limitations of experimental techniques, computational approaches are emerging to complement wet laboratory efforts in elucidating structures of protein assemblies.

**Figure 1:**
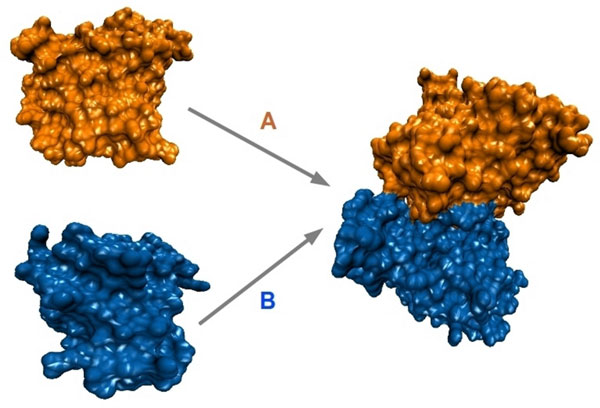
**Protein-protein Docking**. Two unbound units *A *and *B *are docked to form a bound configuration *AB *through rigid-body motion.

When the number of protein units is limited to two, the problem of predicting the biologically-active or native structure formed upon docking of the units onto each other is known as protein docking. This problem is challenging to address in-silico for several reasons. Figure 1 illustrates the docking problem where two unbound units *A *and *B *interacts with each other to form a bound configuration. If no a priori information is available, then the problem requires searching over a space of *N ∗ M *+ 6 dimensions. In this space, *N *and *M *parameters are needed to instantiate the two protein units in different tertiary structures, and 6 parameters are used to represent the rotation and translation components of the spatial arrangement of one unit onto the other, effectively docking the unit designated as moving onto the one designated as the base.

The number of parameters in the general protein-protein docking problem is large, resulting in a high-dimensional search space infeasible for search with systematic approaches. For this reason, many computational approaches elect to reduce the number of parameters by focusing on a sub-problem known as rigid-body docking. In this version of the problem, the tertiary structures of the units are considered to remain the same before and after docking, hence rigid. This effectively does away with the *N *and *M *parameters and presents instead a 6-dimensional SE(3) space, which is the space of (rigid-body) spatial arrangements of the moving unit onto the base unit.

Nowadays, many protein-protein docking software and web servers are available, such as pyDock [4], Haddock [2], Zdock [5], ClusPro [6], PatchDock and SymmDock [7], Combdock [8], Budda [9], Rosetta-Dock [10], SKE-DOCK [11], FiberDock [12], and more. As summaries of their performance in the Critical Assessment of PRedicted Interactions (CAPRI) community-wide experiment are showing, their accuracy is steadily increasing. However, these summaries are also elucidating that no method in particular is best at all target assemblies. Moreover, even the top-performing methods on particular assemblies seem to be able to capture only a fraction of the actual interaction interface [13]. Limited sampling capability, inaccuracy of the energy function used to rank an interaction interface, or a combination of both are often cited as possible reasons for current limitations.

Most docking protocols that do not employ any a priori knowledge about the location of the actual interaction interface in the native dimeric structure follow a similar template that consists of two main stages [14]. In stage one, a large ensemble of dimeric configurations is obtained. Scoring functions are used to increase the likelihood that this ensemble contains configurations near the native structure. The ensemble is reduced in preparation for stage two through either scoring functions that employ more detail or through clustering-based techniques that select a subset of the decoys generated in stage one. The selected decoys are possibly added more structural detail, refined at length through more computationally-intensive energy minimization techniques to make final predictions on which decoys best represent the sought native structure. In some protocols, flexibility is considered to improve the quality of the selected decoys and possibly get closer to the native structure [2]. An important component of the success of this two-stage protocol is the ability of the search algorithm employed in stage one to obtain a relevant ensemble of decoys and not miss the region near the native structure.

The focus of this work is on enhancing sampling of relevant regions in the dimeric configuration space to obtain a diverse decoy of ensemble that can then be analyzed and further refined in energetic detail in the context of ab-initio docking protocols. Towards this goal, we propose here a novel probabilistic search algorithm, HopDock. HopDock samples dimeric configurations that are local minima of some energy function employed to rank interaction interfaces. However, HopDock does not spend its entire computational time operating on the energy surface, which would be computationally demanding. Instead, HopDock elects to operate on the energy surface only to refine configurations that are obtained to align putative interaction interfaces. It is important to note that the definition of a putative interaction interface in this work builds over both the body of geometry-based methods for docking, summarized above, and our own previous work that extends this definition to include evolutionary conservation. In [15,16], we have shown in simple settings of Metropolis Monte Carlo random walks that geometric complementarity and evolutionary conservation are key to narrowing the search space of interest to only a few subspaces shown to include known interaction interfaces in dimers. We build here over this body of work, proposing a more powerful probabilistic search framework that uses this information in a computationally-viable manner. The framework conducts all its global search on regions of SE(3) deemed to contain putative interaction interfaces. Its local search component spends time to further refine interaction interfaces with a simple physics-based energy function.

HopDock is an evolutionary search algorithm and can be considered an algorithmic realization of the Basin Hopping (BH) (or Iterated Local Search) framework [17] (hence, the name HopDock). Our adaptation of the BH framework in HopDock focuses on efficiently navigating the reduced search space (of putative interaction interfaces) to obtain an ensemble of bound configurations corresponding to local minima of a given energy function. Since the focus in this work is on proposal and analysis of effective components in the BH framework for protein docking, the energy function considered here is a simple one consisting of basic physics-based terms.

Our inspiration to build over the BH framework comes from recent findings in the computational structural biology community, including our own preliminary investigation in [18]. Though the BH framework was first proposed to compute structurally-diverse Lennard-Jones minima of small clusters of atomic particles, it has now been shown promising in obtaining low-energy decoy configurations in the context of ab-initio protein structure prediction, where the goal is to predict the structure of a single protein chain in isolation [19-25]. At the core of the BH framework lies a repeated application of a structural perturbation of a configuration followed up by an energetic refinement or minimization of the resulting configuration to obtain a trajectory of low-energy local minima. A Metropolis criterion [26] is used to bias the growing trajectory of consecutive minima towards lower-energy regions of the energy surface.

Our adaptation of the BH framework in HopDock focuses on effective implementations of the perturbation and minimization components that make use of the underlying SE(3) search space. For example, the structural perturbation in HopDock builds over the basic process of aligning geometrically-complementary and evolutionary-conserved regions on the molecular surfaces. The minimization component uses the simple energetic scheme to further optimize a configuration resulting from the structural perturbation.

A preliminary proof-of-concept implementation of this BH-based exploration of dimeric configuration spaces has been presented in [18]. Here we present a more general framework, where we additionally investigate, for instance, the relationship between strength of evolutionary conservation and the ability of the algorithm to capture the correct interaction interface in its ensemble of decoy configurations. Moreover, a detailed analysis over different implementations of the perturbation and minimization components is also carried out in this paper to obtain effective implementations of these components in HopDock. HopDock is benchmarked on a broad and diverse list of protein dimers with known native structures. A detailed comparative analysis places HopDock in the context of other search algorithms used in docking protocols. Our results suggest that HopDock is efficient, competitive, and samples many near-native configurations. These characteristics make it a promising search algorithm to use in the context of docking protocols, particularly if more powerful energy functions are used and if the generated decoys are further selected and refined at greater detail and with more computational resources [27,28].

The rest of this article is organized as follows. We first provide a review of related work in order to place HopDock in context. Details on the different components of HopDock are provided in the Methods section. The Results section evaluates these components on seventeen diverse protein dimers and further compares the result of HopDock to those reported or obtained by current state-of-the-art docking protocols. The Conclusions section provides a discussion and offers promising directions of future research.

### Related work

Current docking methods can primarily be categorized into two approaches, energy-based and geometry-based. Methods like pyDock [4], RosettaDock [10], ClusPro [6], and Haddock [2] take an energy optimization approach. The optimization seeks minima of a defined energy function. If the energy function is sufficiently accurate, near-native configurations will be found among the lowest-energy minima [6,13]. In docking protocols, the process is usually split into two stages. In the first stage, a search is conducted to obtain a large number of low-energy bound configurations. The focus on the size of the ensemble is partially due to the fact that current energy functions are not accurate. Indeed, if only the lowest-energy minimum is maintained in the ensemble, the native structure will certainly be missed by many Ångströms (described in Results section). The size of the ensemble makes it impractical to employ a lot of structural detail and use expensive energy functions. For this reason, typically, the large ensemble is obtained with a simple scoring function. The ensemble is reduced through selection techniques, often relying on structural clustering, to obtain a subset that can be afforded to be optimized in greater structural detail and with more expensive scoring functions in stage two. Computational time can even be devoted in this stage to incorporate some flexibility around detected interfaces in the bound configurations [12].

RosettaDock is a representative of current protocols. The optimization in RosettaDock is carried out over rigid-body orientations and side-chains, followed by continuous minimization. pyDock is another optimization-based server for accurately scoring rigid-body motions. In the first stage, pyDock uses FT-DOCK [29], a Fast Fourier Transform-based docking algorithm, for rigid-body docking. Configurations are then evaluated by their binding energy based on electrostatic and desolvation to obtain a relevant subensemble. As in pyDock, the first stage in ClusPro is performed using a Fast Fourier Transform-based docking algorithm known as DOT [30]. In preparation for stage two, configurations are filtered using a combination of desolvation and electrostatic energies. A clustering algorithm is applied to discriminate against false positives and reduce the set of configurations to near-native structures. Haddock [2] is another example of an energy-based docking protocol that makes use of biochemical data available from NMR to reduce the search space where possible.

Even if computational resources are considered unlimited for optimization, research shows that it remains challenging to design energy functions to accurately score native interaction interfaces [13,31]. For this reason, a group of docking methods take a complementary approach that delays energy considerations to the extent possible.

Instead of conducting the search over a large continuous space, some methods like Budda [9], CombDock [8], PatchDock, SymmDock [7], ZDOCK [5], and LZerD [32] discretize the space by defining geometrically-complementary regions on the molecular surfaces of the units participating in the assembly. The process of searching for arrangements that take one unit over the other then becomes searching for rigid-body transformations that align a region of one molecular surface with a complementary region of the other molecular surface. The main basis of this geometric treatment is that molecules are more likely to interact along geometrically-complementary regions on their surfaces. Convex regions fit better in concave ones, which should produce more stability for docked configurations that superimpose geometrically-complementary regions.

In order to model geometric complementarity, the molecular surfaces of the unbound units need to be analyzed and summarized in terms of geometric properties. Several numerical methods quantize and represent molecular surface with a collection of points, most notably the Connolly [33] and Shuo methods [34]. These methods summarize a molecular surface in terms of "critical" points that contain information on whether the surface region they represent is convex, concave, or saddle. This information is used to consider only rigid-body transformations that align geometrically-complementary regions (such as convex with concave).

The search for geometrically-complementary surface regions is conducted through mainly two approaches. A traditional grid-based shape complementary approach like FTDock [29] identifies grid points surrounding the base unit and the total number of grid points overlapping any grid points corresponding to the moving unit. A more accurate and detailed computer vision-based technique known as Geometric Hashing [35] uses transformation-invariant representations of the molecular surface which allow direct matching. It takes as input a database of objects and a scene in which to find the objects. The algorithm consists of two stages, preprocessing followed by recognition. During the preprocessing stage, some features of the base unit are extracted and hashed into a table. The recognition stage similarly extracts related features from the moving unit and then matches those features to those of the base unit stored in the hash table.

CombDock [8] is based on the technique of Geometric Hashing (GH). In the first stage, CombDock matches geometrically-complementary regions on molecular surfaces. Promising configurations are then subjected to the filtering stage, which uses both geometry and physico-chemical features to identify promising decoys without any use of a detailed energy function. LZerD [32] also uses Geometric Hashing for shape matching in the first step and incorporates a novel geometry-based scoring function using 3D Zernike descriptors in the final step. Multi-LZerD, a recent algorithm for protein assemblies of more than two units [36] uses LZerD for pairwise docking and then relies on a genetic algorithm to sample multimeric configurations. The multimeric decoys are ranked with a physics-based scoring function. Other techniques, like VASP [37], do not carry out explicit docking, but propose useful volumetric-based analysis and representations of surface cavities, clefts, and tunnels to rank and compare binding interfaces.

Due to the implicit discretization of the search space, geometry-based approaches are more efficient but also less accurate than energy-based approaches. Thus, geometry-based approaches are useful to obtain many decoys in an efficient manner. Optimization can be delegated to subsequent stages. Indeed, since their introduction, they have demonstrated that they feasibly produce decoy configurations that then, through further energetic refinement, reproduce biologically-relevant native assemblies [38]. The HopDock algorithm we propose in this paper can be considered to fall in this category, using a simple energy function to refine dimeric configurations found by essentially matching geometrically-complementary regions. In addition, HopDock only explores subspaces of SE(3) of potential interest by narrowing the definition of a putative interaction interface to one that matches both geometrically-complementary and evolutionary-conserved surface regions. We now relate details on the HopDock algorithm.

## Methods

We now relate detail on the HopDock algorithm. As mentioned in the Introduction section, a preliminary proof-of-concept implementation of the BH framework realized in HopDock has been presented in [18]. Our description of HopDock focuses on the overall approach and the novel components added to it in this paper. However, for the sake of completeness, we summarize the components of the preliminary presentation in [18].

In summary, HopDock is based on the BH framework, and the search in it is guided by a simple energy function. The search is conducted over rigid-body transformations that align interfaces that are both geometrically-complementary and evolutionary-conserved. Though these two criteria do not guarantee finding the native interaction interface, they do allow narrowing the search for dimeric configurations to those that align credible interfaces. Geometric complementarity is a well-established predictor for true contact interfaces. Moreover, a detailed analysis in the Results section shows that focusing on evolutionary-conserved regions not only helps finding the correct interaction interface, but more evolutionary-conserved regions are on the native interaction interface than elsewhere on the molecular surface.

Details of the proposed algorithm are presented as follows. First, we define the search space by describing in detail how we use the geometry and evolutionary conservation information to detect rigid-body transformations of interest. Second, we describe a simple energy function that can rank a bound configuration resulting from applications of such a rigid-body transformation. Third, we relate details on how all these elements are incorporated in the proposed HopDock algorithm.

### From molecular surfaces to rigid-body transformations

We now describe how molecular surfaces are analyzed and represented in order to define rigid-body transformations that align chosen surface regions of the units being docked onto each other. Our criteria for choosing certain surface regions are geometric complementary and evolutionary conservation, as detailed below.

#### From molecular surfaces to critical points

The predominant representation of a molecular surface is the Connolly Surface representation [33]. The Connolly method places a probe ball, representing the solvent molecule, tangent to the atoms of the molecule on thousand different locations. For each position of the ball, the point that does not overlap with the van der Waals radii and points facing the inward-surface of the probe becomes part of the molecular surface. The 3D coordinates of each such point are maintained in the Connolly representation of the molecular surface, together with the normal vector and a numeric value to indicate the geometric type of the point. The type ranges from convex, saddle, to concave, and is determined based on the tangency of the probe to the number of atoms on the molecular surface.

The Connolly representation is dense. A sparse representation can be calculated instead [34] that consists of critical points. These are defined as the maxima or minima of a Connolly face of a molecular surface. Critical points are referred to as "caps", "pits," or "belts" to represent the center of gravity of the convex, concave, and saddle surface of the Connolly representation, respectively. The collection of critical points is sufficient and complete to cover key locations of the molecular surface. This sparse representation reduces the total number of points from the high number of points generated by the Connolly surface and so reduce the costs of a geometric treatment in rigid-body docking.

#### From critical points to active critical points: an evolutionary conservation analysis

We now introduce the notion of an active critical point by additionally considering an evolutionary conservation analysis of the molecular surface.

Several studies have shown that molecular regions making up actual interaction interfaces are more evolutionary conserved, probably as a result of higher pressure to retain functional integrity during evolution [39]. Some amino acids are bound to remain more conserved throughout evolution than others if they are involved in an interaction interface. Thus, evolutionary conservation can be a good predictor of the native interaction interface. Several methods [40], [41] now exist for rigorous evolutionary analysis of protein sequences that allow associating evolutionary conservation values with each amino acid of a protein of interest.

The evolutionary analysis method known as Joint Evolutionary Trace (JET) [40], which we employ in this work, allows associating conservation scores with each amino acid of a protein chain. JET relies on multiple sequence alignment and provides rates of conservation known as trace scores. Each amino acid is associated its own trace score in JET. The score is in the [0.0 - 1.0] range corresponding to the least conserved to the most conserved spectrum. We have used iterative JET (iJET), which essentially repeats the JET analysis *n *times to associate a more reliable (average) conservation score with each amino acid. After obtaining such scores, a threshold score *conserve*_*th *_is then used to designate an amino acid as conserved or not conserved. The determination of the value of this parameter and its role in narrowing the focus to the correct interaction interface while not discarding it, is detailed in Results section.

The obtained evolutionary scores can be transferred to critical points. Specifically, a critical point is assigned the conservation score of the surface amino acid closest to it. A critical point with a conservation score greater than *conserve*_*th *_is deemed to be "active" as opposed to "passive." The active/passive designation is inspired by work in [2]. As we detail below, active critical points are used to define surface regions of interest for alignment.

#### From active critical points to active triangles

We now describe how active critical points are used to build active triangles for matching during docking. For any rigid-body motion, a reference frame needs to be defined. In this work, reference frames are defined in terms of active triangles on molecular surface as follows: three critical points are used to define a triangle. At least one of these points has to be active for the triangle to be designated active, as well. First, given the threshold conservation score *conserve_th_*, a critical point is chosen whose conservation score is above the threshold. Let us refer to this point as *p*_1_.

Given *p*_1_, two more critical points (let us refer to them as *p*_2 _and *p*_3_) need to be specified in order to define a triangle. The two other points do not have to be necessarily conserved in our definition of an active triangle, as long as one point (served by *p*_1_) is. The only criteria in selecting *p*_2 _and *p*_3 _is that they have to satisfy angle and distance constraints. Satisfaction of angle constraints is needed so that the points are not collinear and a triangle can indeed be defined. The distance constraints ensure that the resulting triangle is not too small or too large and are based on the ones originally put forth in [38]. A minimum distance of 2.0Å is used so that points are not within this distance of one another (so not on th same van der Waals - vdw - radius of an atom). The maximum distance of 5.0Å ensures the resulting triangle does not cover too much of the molecular surface.

We narrow our focus to unique active triangles in order to limit the number of attempted transformations aligning geometrically-complementary active triangles and avoid redundancy as in [15,16]. A triangle's vertices are first subjected to a lexicographic ordering, which is used to ensure that no two triangles share their first vertex. Further, triangles are hashed by their center of mass in order to ensure that no two triangles share their center of mass. The result of all these constraints is that less than *n *active triangles are defined given *n *critical points.

#### From active triangles to rigid-body transformation

First, one of the units, let's say *A*, is arbitrarily selected as the "base" unit. Therefore, the other unit *B *will be the "moving unit". For each unique active triangle selected from *A*, a matching active triangle is selected from *B*. The features considered for matching two active triangles are only geometric. Suppose the two selected triangles are *tr*_*A *_and *tr_B_*. The rigid-body transformation superimposing triangle *tr*_*B *_over triangle *tr*_*A *_according to 1, will align the monomer *B *on monomer *A*, resulting in a particular dimeric configuration. Thus, a new dimeric configuration is the result of a rigid-body transformation using active triangles.

(1) $$T=tr_{B}^{\prime }*tr_{A}$$

where $tr_{B}^{\prime }$ defines the inverse of reference frame *tr_B_*.

### Energy function

HopDock uses a simple energy function to quickly sample low-energy dimeric configurations to get a broader view of the local minima in energy minimization component. Suppose HopDock has obtained a new dimeric configuration through a rigid-body transformation that aligns two active triangles (one on each unit). We use the following simple energy function to guide the search in HopDock towards configurations that represent minima of this energy function:

(2) $$E=E_{vdW}+E_{electrostatic}+E_{hydrogen-bonding}.$$

The first two terms measure vdw and electrostatic interactions and are based on the CHARMM22 force field [42]. Specifically, the vdw term is based on the standard 12-6 Lennard-Jones potential as follows:

(3) $$E_{vdW}=\underset{atompairs}{\sum }\varepsilon [{\left(\frac{r_{ij}}{d_{ij}}\right)}^{12}-2\times {\left(\frac{r_{ij}}{d_{ij}}\right)}^{6}]$$

where *r*_*ij *_is the atomic radii sum, *E *is the energy well depth derived from CHARM22 [42], and *d*_*ij *_measures the Euclidean distance between atoms *i *and *j*. This energy term penalizes collisions between atoms on one unit and atoms on the other unit in the bound configuration. Atomic pairs (one atom on each unit) that lie not only closer but also farther than an ideal distance (determined by atom types) are also penalized.

The electrostatic term is computed based on Coulomb's law:

(4) $$E_{electrostatic}=\underset{atompairs}{\sum }\frac{q_{i}\times q_{j}}{e\times {d_{ij}}^{2}}$$

where *q*_*i *_and *q*_*j *_are the electrostatic charges of atoms *i *and *j *obtained from CHARM22 [42], *e *is the dielectric constant (vacuum constant 1 is used for this paper), and *d*_*ij *_is the distance between atoms *i *and *j*. The purpose of this term is essentially to penalize atomic pairs that bring similar charges.

The hydrogen-bonding term is calculated through the 12-10 hydrogen potential [43] as follows:

(5) $$E_{hydrogen-bonding}=5\;\times \;{\left(\frac{r_{0}}{d_{ij}}\right)}^{12}-6\;\times \;{\left(\frac{r_{0}}{d_{ij}}\right)}^{10}$$

where *d*_*ij *_measures the Euclidean distance between the interface acceptor atom *i *and interface donor *j*. Here, *r*_0 _= 2.9Å is the idealized distance for hydrogen bonding. The purpose of this term is to reward formation of possible hydrogen bonds between atoms in an interaction interface.

### HopDock: a BH-based algorithm sampling low-energy configurations of dimeric protein assemblies

**Figure 2:**
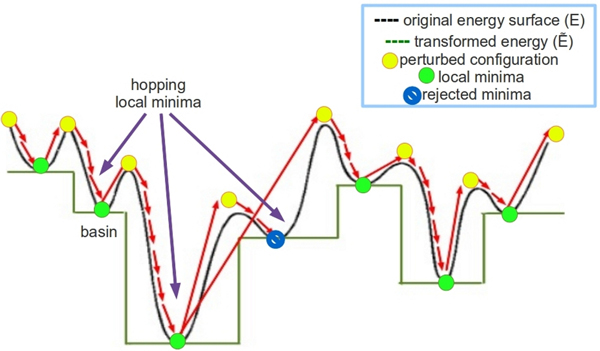
**Overview of Basin Hopping Framework**. Under the BH framework, the energy surface is transformed into a collection of interpenetrating staircases. A trajectory of local minima is obtained consecutively, through iterated applications of a structural perturbation to jump out of a current local minimum and an ensuing local optimization to map to another nearby local minimum.

As in the BH framework, HopDock computes a trajectory of *n *configurations *C*_1_, ..., *C*_*n *_that correspond to minima of a chosen energy function through the BH framework. The general BH framework is illustrated in Figure 2. Starting from a configuration *C*_1 _sampled at random (obtained through a rigid-body transformation aligning sampled geometrically-complementary active triangles), HopDock hops between two consecutive configurations in the trajectory, *C*_*i *_and *C*_*i*+1_, through an intermediate configuration *C*_perturb,i_. A structural perturbation component in HopDock modifies *C*_*i *_to obtain a configuration *C*_perturb,i _that allows escaping the current minimum represented by *C_i_*. The minimization component follows the perturbation. The minimization consists of a series of structural modifications, initiated at *C*_perturb,i_, to obtain a new energetically-refined configuration *C*_*i*+1 _representing the energy minimum nearest to *C*_perturb,i_. The resulting *C*_*i*+1 _configuration is added to the growing trajectory only if it passes the Metropolis criterion. This criterion is based on the difference in energy between *C*_*i *_(the previous minimum in the trajectory) and *C*_*i*+1 _(the current configuration considered a candidate for the new minimum) and an effective temperature serving as a scaling parameter as in *e*^-[*E*(*C*_*i*+1 _)*-E*(*C_i _*)]*/T_e _*^. The objective is for the trajectory of energy minima to converge to lower-energy minima over time. In the Results section, we detail and analyze the effect of different temperature values to select an effective temperature that allows enhancing the sampling of low-energy minima near the native dimeric configuration.

#### Structural perturbation

The perturbation component structurally modifies the current minimum configuration *C*_*i *_by seeking a new rigid-body transformation to obtain *C*_perturb,i_. A naive implementation would not maintain a correlation between the contact interface in *C*_*i *_and that in *C*_perturb,i_. We compare to a naive implementation in Results section and show that preserving some (but not too much) of the contact interface of *C*_*i *_in *C*_perturb,i _is important in obtaining good-quality decoy configurations. We do so by essentially limiting the search for a new contact interface in *C*_perturb,i _to surface regions near the contact interface in *C_i_*. We limit the neighborhood over which new active triangles are sought for matching to obtain *C*_perturb,i_. This is based on studies showing that a perturbation jump of magnitude not too small and not too large results in successful applications of the BH framework for molecular modeling [22-24,44].

Given the current minimum *C_i_*, a dimeric configuration *C*_perturb,i _that preserves some of the good structural characteristics of *C*_*i *_is obtained as follows. Let us refer to the two active triangles that are used to define the rigid-body transformation resulting in the current minimum *C*_*i *_as {*tr_A_*, *tr_B_*} (one from each monomer). The perturbation component samples a new active triangle $tr_{A}^{\prime }$ over the surface of unit *A *uniformly at random in a *d*-neighborhood of *tr_A_*. Here *d *refers to the distance, in angstroms, between the center of mass of *tr*_*A *_and $tr_{A}^{\prime }$. Given the newly sampled $tr_{A}^{\prime }$, a new active triangle $tr_{B}^{\prime }$ is sampled over a *d*-neighborhood of *tr*_*B *_uniformly at random. This process is repeated until a pair of geometrically-complementary active triangles $tr_{A}^{\prime }$ and $tr_{B}^{\prime }$ is found. Once these triangles are obtained, a new rigid-body transformation aligning them is defined (as described above), resulting in the perturbed dimeric configuration *C*_perturb,i_.

Small values of *d *will ensure that *C*_*i *_and *C*_perturb,i _are close in configuration space and so share structural features and parts of their contact interfaces. However, such values may result in no geometrically-complementary active triangles. Large values of *d *increase the probability that a geometrically-complementary pair will be sampled, but they also result in *C*_*i *_and *C*_perturb,i _potentially being far away in configuration space. When that happens, our adaptation of BH in this algorithm degenerates to essentially minimization with random restarts. In the Results section we show the effect of two values of *d *on the magnitude of the perturbation jump and the ability of our algorithm to sample minima near the native configuration. We additionally demonstrate that controlling the value of *d *to some not very large value yields better results than a simple framework that follows up random restart with minimization (where *d *is essentially infinite).

#### Local optimization: energy minimization

The minimization component modifies the perturbed configuration *C*_perturb,i _to obtain a new nearest energy minimum *C*_*i*+1_. The minimization essentially attempts to correct structural features that the perturbation component changed from *C*_*i *_in *C*_perturb,i _and so compute new good features resulting in another energy minimum *C*_*i*+1_. The minimization component in this paper carries out at most *m *consecutive structural modifications, starting with *C*_perturb,i _until *k *consecutive modifications fail to lower energy. Two different implementations are pursued in this paper, depending on how the structural modifications are defined. One straightforward implementation is to define each of these modifications essentially as versions of the perturbation component, but with smaller *d*. The purpose of making *d *small is so that the minimization brings *C*_perturb,i _to the nearest local minimum and not to some random point in the configuration space.

Our analysis shows that it can be hard to find small values of *d *that will still allow finding geometrically-complementary active triangles. Therefore, this implementation is not effective, as it tends to make large jumps in configuration space all the while attempting to lower energy. Therefore, a new implementation is pursued for the minimization component. This implementation essentially samples new rigid-body transformations directly, rather than through active triangles, in a continuous neighborhood of an initial transformation.

A rigid-body transformation is represented as 〈*t*, *u*, *θ*〉, where *t *specifies the translation component, and 〈*u*, *θ*〉 specify the orientation component in an axis-angle representation (implemented here through quaternions). In each modification in the minimization component, a new random transformation is sampled in the neighborhood of the transformation representing the configuration resulting from the previous modification. The translation and rotation components are sampled individually. A new translation component is sampled in a *δ*_*t *_neighborhood of *t*. A new rotation component is obtained by sampling a new axis *u *rotating around the axis *u *by a sampled angle value *δ_ϕ_*; a new angle is obtained by sampling in a *δ*_*θ *_neighborhood around *θ*.

The implementations we propose for the minimization component do not seek to identify the true basin of a local minimum. The depth of the exploration is determined by the parameter *m *in the minimization. Given that the decoys need to be low-energy but can be refined in detail at a later stage, this approximate definition of a local minimum is sufficient. For this reason, the minimization component employs a simple energy function.

## Results and discussion

The organization of this section is as follows. The implementation details and the protein systems employed here for validation of HopDock are described first. Second, an analysis of the distribution of evolutionary-conserved regions on the molecular surface in finding the true interaction interface is presented in the next section. The next few sections provide a detailed analysis on how values of different parameters in HopDock have been chosen. The parameters analyzed here are the evolutionary conservation threshold, the effective temperature *T*_*e *_employed in Metropolis Criterion, the perturbation distance *d *in the perturbation component, and the translation distance *t *in the minimization component. In the following section an analysis has been performed to investigate the relationship between the lower energy values to the near-native structures. A detailed comparative analysis on the attainment of the known native configuration for the proteins systems studied in this work to other state-of-the-art docking protocols is provided in the last section.

### Experimental setup

#### Implementation details

HopDock was run on a 3GHz of Opteron Processor with 4GB of memory to generate 10, 000 dimeric configurations per protein system considered. A detailed analysis of HopDock was conducted on 5, 000 to 20, 000 configurations. Results obtained with r 10, 000 configurations were found to be representative, so the analysis presented below is over 10, 000 sampled configurations. Depending on the size of the protein systems under investigation, obtaining this number of configurations takes anywhere from 1 - 12 hours on one CPU.

#### Performance measurements

Our analysis employs least Root-Mean-Square-Deviation (lRMSD) to the known native dimeric structure to determine the quality of a generated configuration. lRMSD is a widely accepted performance measurements in docking methods, reported in units of Ångström (Å). RMSD is a measure of the average atomic displacement between two configurations, say x and y, under comparison and is calculated as follows:

(6) $$\sqrt{\frac{1}{N}\overset{N}{\underset{i=1}{\sum }}||x_{i}-y_{i}|{|}^{2}}$$

lRMSD refers to the minimum RMSD over all possible rigid-body motions of one configuration relative to the other. A value between 2 and 5Å is considered to be indicative of a configuration being highly similar to the known native structure, and the configuration is deemed near-native. We use lRMSD here not only to determine the proximity of dimeric configurations generated by HopDock to the known native structure but also to analyze the proximity of configurations to each other in the trajectory generated by HopDock.

#### Protein systems of study

We have selected seventeen different dimers with known native structures obtained from PDB as our systems of study. These dimers, listed in Table 1 are chosen because they vary in size, functional class, and have been studied by other docking methods, as well. Table 1 lists the PDB ID of the known native structure of each dimer in column 1, the size of each unit in a dimer in terms of number of atoms in column 2, and the known functional classification obtained from PDB in column 3. Systems that are CAPRI targets are marked with an asterisk in column 1.

**Table 1:** Protein systems of study.

PDB ID (Chains)	Size(Number of Atoms)	Functional Classification
1C1Y (A,B)	1376, 658	Signaling Protein
1DS6 (A,B)	1413, 1426	Signaling Protein
1TX4 (A,B)	1579, 1378	Complex(gtpase Activatn/proto Oncogene)
1WWW (W,Y)	862, 782	Nerve Growth Factor/trka Complex
1FLT (V,Y)	770, 758	Complex (growth Factor/transferase)
1IKN (A,C)	2262, 916	Transcription Factor
1IKN (C,D)	916, 1589	Transcription Factor
1VCB (A,B)	755, 692	Transcription
1VCB (B,C)	692, 1154	Transcription
1OHZ* (A,B)	1027, 416	Cell Adhesion
1T6G* (A,C)	2628, 1394	Hydrolase Inhibitor
1ZHI* (A,B)	1597, 1036	Transcription/replication
2HQS* (A,C)	3127, 856	Transport Protein/lipoprotein
1QAV (A,B)	663, 840	Membrane Protein/oxidoreductase
1G4Y (B,R)	682, 1156	Signaling Protein
1CSE (E,I)	1920, 522	Complex(serine Proteinase Inhibitor)
1G4U (R,S)	1398, 2790	Signaling Protein

Details are provided on the dimers selected in this study. PDB ID of known native structure along with chains in bracket is shown in column 1. Column 2 lists the number of atoms per unit in a dimer, and column 3 shows the known functional classification of each dimer. CAPRI targets are marked with an asterisk.

### Evolutionary conservation analysis preserves native interface

The role of evolutionary conservation in finding the true contact interface has been demonstrated in other studies [45,46]. The work in [45] shows good correlation between sequence conservation and inclusion of conserved surface regions in the interaction interface. The analysis in [45] was expansive, classifying 265 proteins into different functional categories and measuring the correlation between conservation and inclusion of conserved regions in the interaction interface through Matthews' correlation coefficient (MCC) [47]. Analysis of MCC values allowed concluding that interaction interfaces in signal proteins and enzymes was particularly conserved. A larger dataset of 2646 protein interfaces was analyzed in [46]. The study concluded that not only there are highly conserved surface regions on the majority of proteins, but also in most proteins these regions are more likely to be found on interaction interfaces than on the rest of the molecular surface.

We conduct here a similar analysis to that in [46] to investigate the relationship between the known interaction interface and evolutionary-conserved regions based on our working definition of active critical points (described in Methods section). The goal is to uncover any correlation between high evolutionary conservation score and the true contact interface. We point out that our analysis is confined to the 17 systems studied in this work, 8 of which were specifically chosen due to their inclusion in the study in [46]. We measure two different ratios, *R*_*interface *_and *R*_*rest *_on each of the known native dimeric structures of the systems studied here. *R*_*interface *_is the ratio between the number of conserved critical points on the known interaction interface to the total number of critical points on that interaction interface. *R*_*rest *_is 1 *- R_interface_*. Two critical points, one from the molecular surface of unit A and the other on that of unit B in known native dimeric structure, are considered to be in contact and thus in the true interaction interface if their Euclidean distance is no higher than 5Å. This distance threshold is commonly employed in other work [2,5].

**Figure 3:**
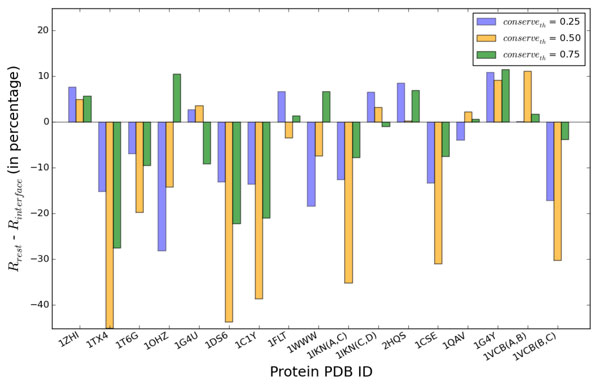
**Analysis of Evolutionary Conservation**. Column diagram shows the *R_rest _- R*_*interface *_difference on each of the seventeen dimers. Three different conservation thresholds are considered and results for each are shown in different colors. Negative percentages indicate the interface is more conserved than the rest of the molecular surface.

Each of the ratios described above is measured as a percentage, and the difference between them, *R_rest _- R_interface_*, is plotted in Figure 3 in column diagram format. A negative value indicates that the interaction interface is more conserved than the rest of the surface, as more of the active critical points fall in it rather than elsewhere on the molecular surface. Results are shown for three different conservation thresholds in {0.25, 0.50, 0.75} in different colors to visualize the effect that varying the conservation threshold has on the distribution of conserved critical points.

The results in Figure 3 allow concluding that in 10 of the 17 dimers, the distribution of conserved critical points is heavily concentrated on the true interaction interface as opposed to the rest of the molecular surface. In the context of employing this information for docking, this result means that more rigid-body motions will focus on matching regions on the actual interaction interface than elsewhere on the molecular surfaces. On the rest of the dimers, Figure 3 shows that the difference is not too large, which means HopDock will not spend a large portion of its time on matching regions elsewhere on the molecular surface rather than on the known interaction interface.

### Analysis of different parameter values employed in HopDock

Here we investigate in detail the effect that varying values of certain parameters in HopDock has on the quality of the ensemble of sampled dimeric configurations. The parameters we investigate are the conservation threshold and its effect on the size of the search space, the effective temperature employed in the Metropolis criterion and its effect on the energetic and structural quality of sampled configurations, and the perturbation distance in the perturbation component and translation distance in the minimization component and their effect on the overall quality of the sampled ensemble.

#### Analysis of evolutionary conservation threshold

Analysis on the choice of the conservation threshold, *conserve_th_*, is presented in Table 2 on three selected systems that considers the same three different thresholds as in Figure 3. The results of this table are obtained through our previous work [15,16]. The analysis focuses on showing the effect of different evolutionary conservation thresholds on the number of active triangles and essentially on the lowest lRMSD to native that can be achieved. Column 1 indicates the PDB ID of these three systems and their chains in brackets. Three conservation thresholds in {0.25, 0.5, 0.75} are employed to define three sets of active triangles and are reported in column 2. Column 3 shows the number of active triangles defined on the molecular surface of the base/reference unit under each conservation threshold. For each system, three sets of dimeric configurations, one for each threshold, are then obtained from our previous work. The lowest lRMSD to the experimentally-determined native structure from these dimeric configurations is recorded for each set and these values are reported for each system in column 4. The last column shows *R_rest_-R*_*interface *_recorded for each conservation threshold as from Figure 3.

**Table 2:** Conservation threshold analysis.

PDB ID	Threshold	Nr. Triangles	lRMSD (Å)	*Rrest - Rinterface*
1FLT (V,Y)	0.25	2417	2.06	6.67
	0.50	2338	1.12	-3.50
	0.75	2080	1.03	1.27

1WWW (W, Y)	0.25	2900	2.29	-18.37
	0.50	2911	2.24	-7.45
	0.75	2854	2.60	6.62

1C1Y (A,B)	0.25	3385	1.89	-13.65
	0.50	3325	1.30	-38.64
	0.75	3306	1.45	-21.01

Table shows the effect of three different conservation thresholds 0.25, 0.5, and 0.75 on the number of active triangles and lowest lRMSD to native structure.

Table 2 highlights a few results. First, the number of active triangles decreases as the conservation threshold increases. This is expected, since a higher threshold limits the number of active critical points which in turn limit the number of active triangles. Second, the lowest lRMSD generally decreases as the conservation threshold increases, but the effect on lRMSD depends on how much of the conserved critical points under each conservation threshold remain on the true interaction interface as opposed to elsewhere. These results, combined with our analysis on the distribution of conserved critical points on the molecular surface above, allow us to conclude that a conservation threshold of 0.5 is a reasonable compromise between maintaining a smaller number of active triangles, thus reducing the size of the search space, while retaining the known interaction interface. For this reason, the rest of our experiments employ *conserve*_*th *_= 0.5 as conservation threshold.

#### Analysis of effective temperature

The effective temperature *T*_*e *_in the Metropolis criterion affects the acceptance probability *e^-δETe ^*with which an energetic increase *δE *is accepted in the trajectory. A higher temperature increases the probability to accept an energy increase between two consecutive configurations in the trajectory than a lower temperature. A high temperature will allow HopDock to make large jumps in the energy surface, effectively degenerating to a random restart search where there is no correlation between two consecutive configurations in the HopDock trajectory. On the other hand, a low temperature may provide too strong a bias and not allow HopDock to accept temporary energetic increases to potentially cross energy barriers needed for convergence to deeper local minima over time.

In [18], we compare various effective temperatures and their effect on two measures of performance, lowest lRMSD to the native structure and percentage of configurations with lRMSD *<*5Å to the native structure. The analysis focuses on two representative temperatures, *T*_0_, a representative of a medium temperature and *T*_1_, a representative of a lower temperature. *T*_1 _allows accepting an energy increase of 2 kcal/mol with probability of 0.39, allows accepting that same energy increase with a lower probability of 0.16. The analysis presented in [18] allows concluding that *T*_0 _allows HopDock to generate slightly more near-native configurations than *T*_1 _does. The lower temperature *T*_1 _allows achieving lower energies, as expected, but these do not necessarily translate to near-native configurations when dealing with inaccurate energy functions. Based on this analysis, we have chosen the higher temperature *T*_0 _to allow more energetic diversity for the rest of the experiments in the Metropolis criterion that guides the acceptance of local minima sampled by HopDock.

#### Analysis of perturbation and minimization distance

The next experiment compares different implementations of the perturbation distance *d *described in the Methods section. The first implementation essentially allows testing the efficacy of random restart. In this implementation, a geometrically-complementary pair of triangles is sampled uniformly at random, and the resulting transformation is applied to obtain a new perturbed configuration. This implementation essentially refers to the case when *d *= *∞*, since it employs no knowledge of the pair of triangles that were aligned to obtain the previous minimum *C_i_*. In the second and third implementations, *d *is controlled and set to 7 and 5Å, respectively. A smaller value of *d *makes it very hard to find geometrically-complementary triangles on the molecular surfaces.

**Figure 4:**
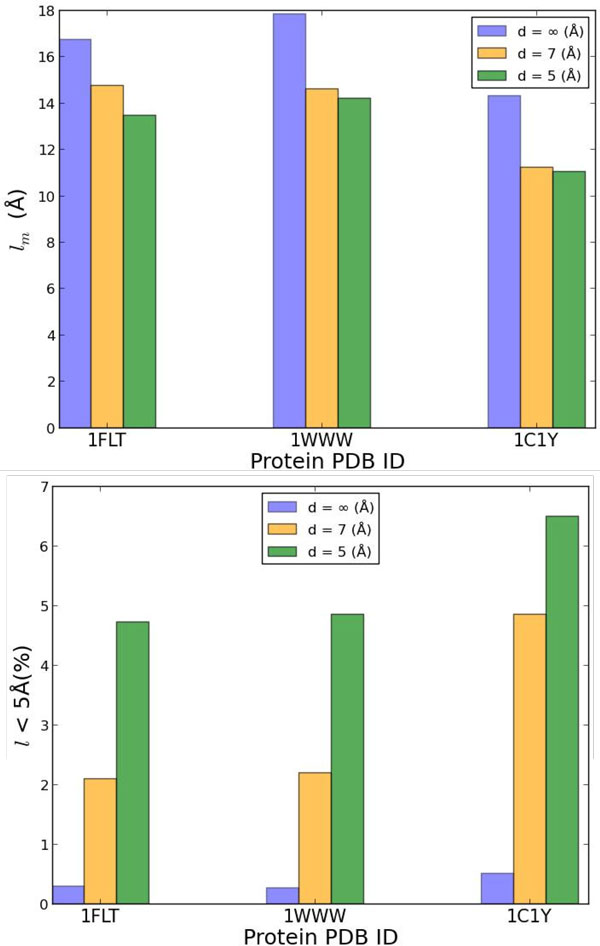
**Analysis of Effect of Perturbation Distance**. The effect of perturbation distance on three representative systems is shown here to summarize the detailed analysis in [18]. Three different values of *d *are considered, *∞*, 5Å and 7Å, color-coded in different colors. The bars in the top pannel (a) show the median *l*_*m *_of the *l *distribution of lRMSDs between two consecutive perturbed configurations. Those in the bottom pannel (b) show the percentage of consecutive distances within 5Å of each-other.

Our preliminary investigation in [18] compares these three different implementations in terms of various statistics. First, the distance in terms of lRMSD between two consecutive perturbed configurations, *C*_perturb,i _and *C*_perturb,i+1 _is recorded to obtain a measure of the magnitude of the perturbation jump in each setting. Let us refer to this distance as *l*. The median over these distances over all perturbed configurations obtained in the process of running HopDock with each of the three implementations of the perturbation component, *l*_*m *_is also tracked. The actual distribution of these distances is investigated in more detail by tracking the percentage of distances in the 0-5Å and 5-10Å range. The effect of controlling *d *is also evaluated in terms of the quality of the overall ensemble of sampled minima by measuring the lowest lRMSD to the native structure and the percentage of minima configurations with lRMSD to the native structure less than 5Å. All these measurements are compared across the three settings of *d *∈ {*∞*, 7, 5}Å, and our detailed analysis in [18] allows concluding that *d *= 5Årepresents an optimal setting. We highlight the most salient conclusions of that analysis in Figure 4. We show in Figure 4(a) that controlling *d *allows controlling the median distance *l_m_*. Additionally, Figure 4(b) shows that lowering *d *also increases the number of consecutive perturbed configurations with *<*5Å of each-other. The full analysis in [18] shows this is also true for perturbed configurations with *<*10Å lRMSD of each-other, and that lowering *d *does not impact the number of minima with low lRMSDs to the native structure negatively. For this reason, we employ *d *= 5Å in the rest of our experiments below.

Finally, our preliminary investigation in [18] also pitches different implementations of the minimization component against one another. In all implementations, *m *= 100 and *k *= 20 (i.e., the minimization trajectory initiated from a perturbed configuration is at most 100 steps long and can terminate earlier, if 20 consecutive steps all fail to lower energy). In the Methods section we explain that the consecutive modifications pursued in the minimization component to lower interaction energy can be considered versions of the perturbation component with *d *= 5Å. However, these result in large moves in the minimization component and do not ensure that a perturbed configuration will be projected to a nearby local minimum rather than some uncorrelated configuration in the search space. Our analysis suggests that this implementation is less effective. The results below show the effect of three other implementations, which sample rigid-body transformations directly, rather than through active triangles, in a continuous neighborhood of the rigid-body transformation in the previous configuration in the minimization trajectory. These implementations use the same thresholds of *δ_ϕ _*= 10*° *and *δ*_*θ *_= 30*°*. What varies is the translation distance threshold *t*, which takes values in {1.5, 2.0, 2.5}Å. We decide to focus on varying *t *rather than the orientation, as the translation distance is expected to have a more dramatic effect on changing the contact interface. The goal of this analysis is to determine whether making small moves during minimization, which increases the probability of actually populating the minimum nearest to *C*_perturb,i_, has any effect on the distance between a perturbed configurations and its nearby minimum as well as on the overall quality of sampled local minima.

In summary, the analysis in [18] compares these implementations in terms of various statistics. First, the distance between *C*_perturb,i _to *C*_*i *_sampled by HopDock is recorded, referred to as *i*. The distribution of distances is tracked and compared across the various settings in terms of its median *i*_*m *_and the percentage of distances in the 0 - 5Å and 5 - 10Å range. The overall effect on the quality of the ensemble of configurations is also measured in terms of the lowest lRMSD to the native structure and the percentage of minima configurations with lRMSD *<*5Å to the native structure. The analysis in [18] suggests that varying *t *in this range does not seem to result significant differences in the measured statistics. However, comparing the lowest lRMSD to the native structure and the overall number of minima within 5Å of the native structure shows that a translation distance *t *= 1.5Å provides a good compromise. For this reason, the rest of our experiments below use *t *= 1.5Å.

### Analysis of relationship between energy and proximity to native structure

**Figure 5:**
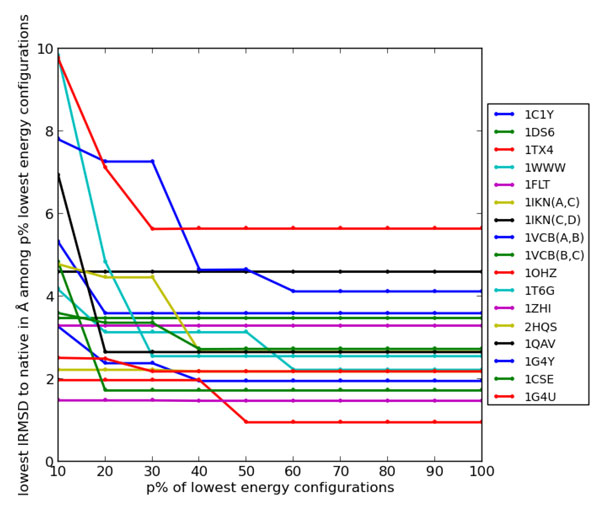
**Analysis of Lowest Energy Values**. Lowest lRMSD from native structure is shown for each reduced Ω_*p *_ensemble on all 17 protein systems. Ω_*p *_contains the *p*% lowest-energy configurations generated by HopDock. Results for each protein system are shown in different colors.

Based on the above analysis, an ensemble of 10, 000 dimeric configurations is obtained for each of the 17 protein systems using HopDock with *conserve*_*th *_= 0.5, *T*_*e *_= *T*_0_, *d *= 5Å, and *t *= 1.5Å. Here we investigate the extent to which an energetic reduction scheme that reduces the sampled ensemble Ω based on low energies is able to retain near-native configurations with low lRMSDs to the known native structure. For this purpose, we define a variable *p *to track the % of configurations with lowest energies retained in a reduced ensemble Ω_*p*_. *p *is varied from 10 - 100% increments of 10. Ω_*p *= 100 _means that the entire ensemble Ω is retained. Ω_*p *= 10 _means that only the 10% of the configurations with lowest energies are retained. Figure 5 plots the lowest lRMSD to the native structure over configurations in each reduced ensemble Ω_*p *_as *p *is varied. This is shown for each of the 17 protein systems. Figure 5 shows that the lowest lRMSD obtained by HopDock over the entire ensemble is retained even when *p *= 40 - 50% for most protein systems. This effectively means that near-native configurations are not discarded if focusing only on those with energies below the mean. For many systems, the lowest lRMSD does not change significantly even when only p is further reduced. This result is encouraging, particularly, if HopDock is considered as a configuration sampling technique to be employed in docking protocols. Even this coarse reduction by energy ensures that near-native configurations will be present in the reduced ensemble and can be further refined by docking protocols to improve their proximity to the native structure.

### Comparative analysis of HopDock to other existing methods

**Figure 6:**
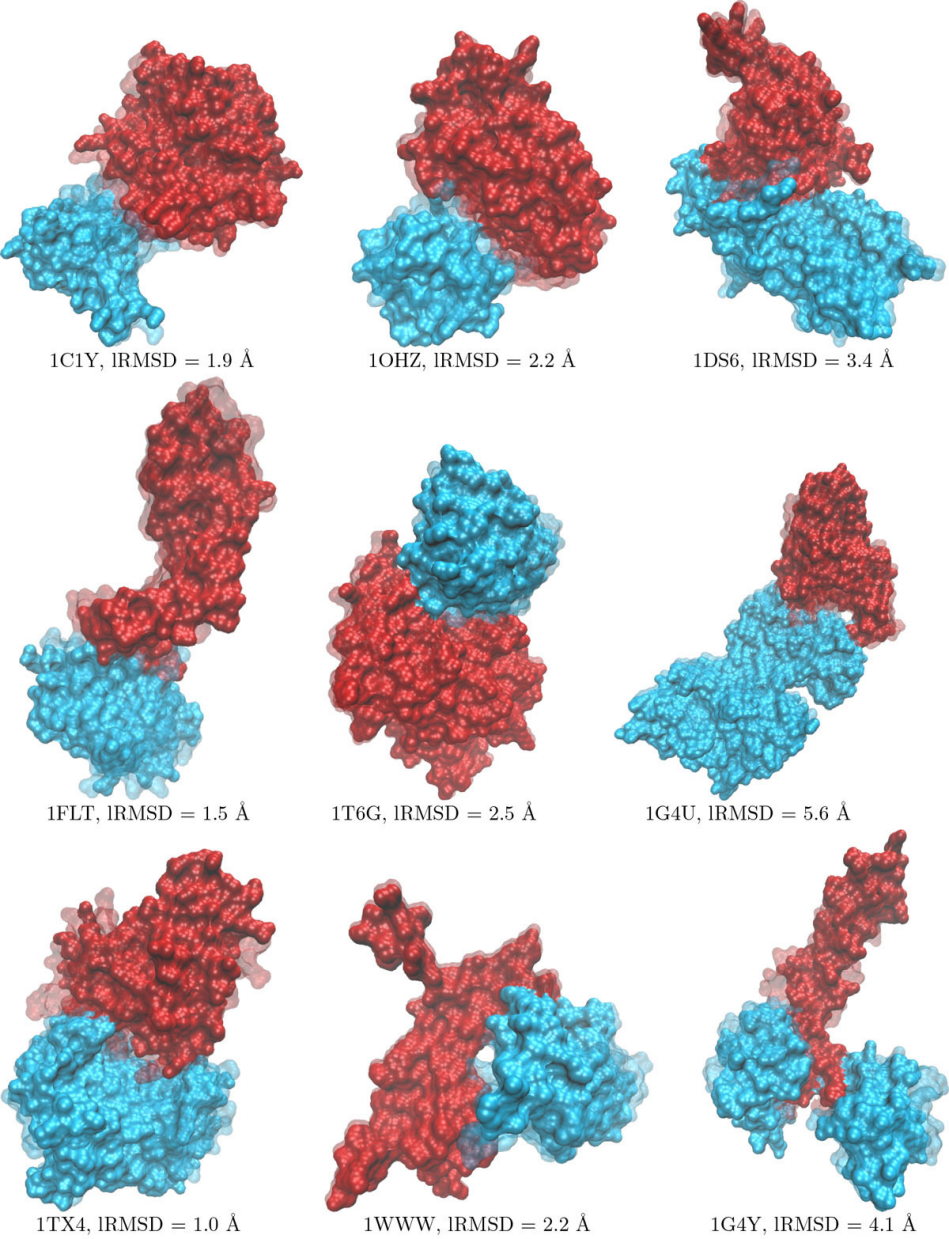
**Analysis of Lowest lRMSD to Native Structures**. Nine systems are selected to draw the lowest-lRMSD configuration obtained by HopDock. This configuration is drawn in opaque, with chains in blue and red. The native structure, over which the lowest-lRMSD configuration is superimposed to highlight structural differences, is drawn in transparent. The actual lRMSD between the two is shown below. Visualization is obtained through VMD [50].

Table 3 shows the results obtained when applying HopDock (with the parameter values and implementations above) on the seventeen protein systems studied here. Columns 1-2 relate details on these dimers in terms of the PDB ID of the known native structure of the dimer and size (total number of atoms). Column 3 shows the lowest lRMSDs to the native structure as reported by Budda in [9], which uses geometric complementarity to match regions for docking and relies on clustering and some short energetic refinements of top clusters. Column 4 shows again for reference the lowest lRMSD obtained from our previous work which relies on exhaustive matching of active geometrically-complementary triangles [16]. Columns 5 and 6 show the lowest lRMSDs obtained on each dimer with the pyDock [4] and ClusPro [6] servers, respectively. The methods selected for the comparison are representative of geometry- and energy-based methods commonly used by docking protocols. Column 7 reports the lowest lRMSD obtained by the HopDock (the lowest-lRMSD configuration is shown superimposed on the native structure for 9 systems in Figure 6).

**Table 3:** Final Results of HopDock presented in this work.

PDB ID (Chains)	Size	**Budda **[9]**(Å)**	**Prev. **[16]**(Å)**	**pyDock **[4]**(Å)**	**ClusPro **[6]**(Å)**	HopDock (Å)
1C1Y (A,B)	2034	1.2	1.3	10.4	7.2	1.9
1DS6 (A,B)	2839	1.2	1.8	0.8	1.7	3.4
1TX4 (A,B)	2957	1.4	2.4	18.5	4.7	1.0
1WWW (W,Y)	1644	11.4	2.2	18.2	17.2	2.2
1FLT (V,Y)	1528	1.5	1.1	2.8	4.7	1.5
1IKN (A,C)	3178	1.2	2.0	20.1	19.7	2.2
1IKN (C,D)	2505	2.0	2.0	16.7	20.9	4.6
1VCB (A,B)	1447	0.7	2.1	1.4	1.9	3.6
1VCB (B,C)	1846	1.3	1.3	22.7	1.9	1.7
1OHZ (A,B)	1443	1.8	1.7	7.5	3.3	2.2
1T6G (A,C)	4022	1.6	2.5	0.1	14.8	2.5
1ZHI (A,B)	2633	25.3	1.7	23.8	24.1	3.3
2HQS (A,C)	3983	29.1	2.2	15.2	16.6	2.6
1QAV (A,B)	1503	1.4	1.0	9.6	1.7	2.6
1G4Y (B,R)	1838	0.8	2.3	26.2	1.9	4.1
1CSE (E,I)	2442	0.7	1.5	13.2	1.1	2.7
1G4U (R,S)	4188	1.0	2.2	27.6	16.1	5.6

A comparative analysis has been performed on all seventeen dimers. For the comparison, two geometry-based (Budda [9] and work in [16]) and two energy-based methods (pyDock [4] and ClusPro [6]) have been chosen. Lowest lRMSD to native structure is reported for Budda in column 3, work in [16] in column 4, pyDock in column 5, ClusPro in column 6, and HopDock in column 7.

Table 3 shows that HopDock obtains low lRMSDs to the native structure on each dimer. These lRMSDs are comparable to the geometric-based Budda method and our previous work in [16] on most protein systems. In few cases, our previous work, which relies on exhaustive sampling of geometrically-complementary active triangles, achieves slightly lower lowest lRMSDs than HopDock. This is largely due to the fact that a very large number of dimeric configurations, 100, 000 to 700, 000, depending on the size of the proteins are sampled in [16]. It is indeed encouraging to obtain similar lRMSDs, and even lower in some cases, with HopDock, which samples only about 10% of the configurations in [16]. The comparison of HopDock to two energy-based methods, pyDock and ClusPro, allows us to obtain a more comprehensive view of the results obtained by HopDock. In comparison to PyDock and ClusPro, the performance of HopDock in terms of proximity to the known native structure is better or comparable. This is particularly the case as the size of the protein system grows. This result is expected, as energy-based optimization methods operate on a large search space, whereas HopDock focuses on potentially-relevant contact interfaces through its combination of geometry and evolutionary conservation.

#### Comparative analysis of computing time and power

The above results are promising and suggest that HopDock is an important first step into a multi-stage docking protocol. Here we provide a broader picture by comparing HopDock to two established docking servers, pyDock [4] and ClusPro [6]. The purpose of this analysis is to better gage how HopDock compares to these servers, even though we are fully aware that the implementations in these servers are tuned and optimized, and that the methods in these servers use different search approaches and sophisticated energy functions. To get a sense of the resources available to HopDock as compared to these other serves, Table 4 summarizes number of processors, processing speed, memory, and average CPU time used by each method. Table 5 shows the time that each of these servers, including our own algorithm, HopDock, takes on each of the 17 systems studied here. This table shows that ClusPro is faster on most systems, but HopDock, though not fine tuned, achieves comparable running times to both servers. Taken together, these results suggest that HopDock is a promising search algorithm that ca be used in the first stage by docking protocols.

**Table 4:** Summary of the computing power of different docking protocols.

Protocols	Number of Processor	Processing Speed (GHz)	Memory(GB)	Average CPU Time (Hours)
pyDock	2 Nodes(16 core each)	2.4	65	3-5
ClusPro	16	1.3	32 (shared)	4
HopDock	2 (Core 2 Duo)	3.00	4	3-5

Summary of computing power of different protein-protein docking protocols is presented to place HopDock in context.

**Table 5:** Timing comparison.

PDB ID (Chains)	HopDock (HH:MM)	**pyDock **[4]**(HH:MM)**	**ClusPro **[6]**(HH:MM)**
1C1Y (A,B)	04:00	01:30	00:53
1DS6 (A,B)	06:26	02:00	01.30
1TX4 (A,B)	10:42	02:30	01:00
1WWW (W,Y)	03:12	01:00	00:53
1FLT (V,Y)	02:36	00:30	00:53
1IKN (A,C)	06:00	01:30	01.24
1IKN (C,D)	03:54	00:18	01.24
1VCB (A,B)	01:04	00:33	00.54
1VCB (B,C)	01:36	01:08	00:58
1OHZ (A,B)	00:57	02:30	01:00
1T6G (A,C)	11:04	04:00	00:59
1ZHI (A,B)	03:17	04:45	00:59
2HQS (A,C)	12:07	01:00	01:00
1QAV (A,B)	01:05	01:30	00:40
1G4Y (B,R)	03:13	05:29	00:59
1CSE (E,I)	02:48	02:00	00:38
1G4U (R,S)	09:19	01:14	01:26

Timing comparison (HH:MM) of HopDock to other web servers is presented on each of the seventeen systems studied here.

## Conclusion

We have presented here HopDock, a novel rigid-body protein docking algorithm based on the BH frame-work, to efficiently generate low-energy decoy configurations for dimeric systems. The algorithm conducts its search over the SE(3) space of rigid-body transformations but narrows its focus to regions corresponding to transformations aligning evolutionary conserved geometrically-complementary regions on molecular surfaces. The incorporation of evolutionary information reduces and simplifies the search space over which the structural perturbation in HopDock selects the dimeric configurations for minimization component. Since HopDock is a decoy sampling algorithm, a simple energy function has been employed to reduce the time complexity of the minimization component and measure the performance of HopDock. A detailed analysis of evolutionary conservation and different components of HopDock has been presented in this work. This analysis shows that HopDock produces many near-native configurations in the decoy ensemble it samples. A detailed comparative analysis shows that the algorithm is competitive with other state-of-the-art protocols. This suggests that HopDock is a promising decoy sampling algorithm to be incorporated in a docking protocols.

There are several further directions for future research. One involves taking into account additional criteria beyond evolutionary conservation to predict interaction interfaces [48,49]. Pursuit of more sophisticated energy functions used by state-of-the-art docking protocols is another direction. We also intend to pursue different implementations, especially for the minimization component. While the perturbation component can focus on expediently obtaining configurations at a low-resolution level of detail (exploring the SE(3) space of rigid-body transformations), the minimization can add more detail to project a low-resolution configuration onto a nearby minimum of a more detailed energy surface. Combined with clustering and further refinement of top-populated clusters, the combination of a geometric and energetic treatment proposed here promises to result in an effective docking protocol. Wider sampling through population-based versions of the BH framework and incorporation of fluctuations on and nearby interaction interfaces will also be considered to improve the quality of decoy ensembles.

## Competing interests

The authors declare that they have no competing interests.

## Authors' contributions

IH suggested the methods and the performance study in this manuscript and drafted the manuscript. AS guided the study, provided comments and suggestions on the methods and performance evaluation, and improved the manuscript writing.

## Declarations

The publication costs for this article were funded by the corresponding author AS through NSF Grant No. 1144106.

This article has been published as part of *Proteome Science *Volume 11 Supplement 1, 2013: Selected articles from the IEEE International Conference on Bioinformatics and Biomedicine 2012: Proteome Science. The full contents of the supplement are available online at http://www.proteomesci.com/supplements/11/S1.
